# Evaluation of *in ovo* feeding of low or high mixtures of cysteine and lysine on performance, intestinal morphology and physiological responses of thermal-challenged broiler embryos

**DOI:** 10.3389/fphys.2022.972041

**Published:** 2022-09-02

**Authors:** O. I. Ajayi, O. F. Smith, A. O. Oso, O. E. Oke

**Affiliations:** ^1^ Department of Animal Physiology, Federal University of Agriculture, Abeokuta, Nigeria; ^2^ Department of Animal Nutrition, Federal University of Agriculture, Abeokuta, Nigeria

**Keywords:** in ovo, heat stress, cysteine, lysine, performance, thermotolerance

## Abstract

The objective of this study was to evaluate the effect of in ovo feeding cysteine, lysine or their combinations on the perinatal and post-hatch physiological responses of broiler embryos exposed to heat stress during incubation. A total of two thousand fertile eggs of broiler breeders (Ross 308) flock (at 38 weeks of age) were used for this study. In the first 10 days, the eggs were incubated using the conventional protocol of relative humidity and temperature of 55% and 37.8°C respectively. From day ten onward, the temperature was increased to 39.6°C for 6 h per day. On day 17.5, 1,500 eggs with the evidence of living embryos were randomly selected and assigned to 6 treatments having five replicates of 50 eggs each. The treatments were: un-injected eggs (UI), eggs injected with only 0.5 ml distilled water (DW), 3.5 mg/egg cysteine (CY), 2mg/egg lysine (LY), 3.4 mg cysteine+2 mg lysine (CLH) and 1.7 mg cysteine+1 mg lysine (CLL). On day 21, the hatchability, anatomical characteristics, chick quality and the antioxidant status of the chicks were evaluated. During the post-hatch phase, data were collected on the haematology, biochemical parameters, growth performance and intestinal morphology of the birds. The results revealed that the hatchability of CY chicks was higher (*p* < 0.05) than in the other treatments, while the lowest values were recorded in CLH. The hatching muscle of the chicks of CLL was similar to those of CY but higher (*p* < 0.05) than the others. The MDA of DW and UI chickens was similar and higher than birds in the other treatment groups. The serum SOD of CLL birds was comparable to that of CY but higher than the values recorded in the other treatments. The final weights of CLL chickens were similar to those of LY but significantly higher (*p* < 0.05) than those of the other treatments. The duodenal villus heights of the birds of CLL were higher than those of the other treatment groups, whereas the villus height of the birds of CLH was higher than those of UI, DW and CY. Overall, *in ovo* feeding of cysteine alone improved the hatchability of thermally-challenged broiler embryos. In contrast, a low-dose mixture of cysteine plus lysine improved the post-hatch growth performance.

## 1 Introduction

Global warming is increasing Earth’s average surface temperature, causing livestock to suffer heat stress (Lara and Rostagno, 2013), resulting in poor productivity (Oke et al., 2017; Oke, 2018). This situation is exacerbated in chickens as they have no sweat glands and are susceptible to thermal stress. Chickens under heat stress exhibit increased body temperatures, oxidative stress and metabolic amino acid alteration (Brosnan and Brosnan, 2006; Chowdhury et al., 2014; Oke et al., 2021a; Chowdhury et al., 2021; Kpomasse et al., 2021). In addition to serving as protein building blocks, amino acids act as regulators of gene expression and mediators of phosphorylation of protein cascades. They also regulate growth and body temperature, control food intake, and influence behaviour. Findings have revealed that amino acids could serve as heat stress biomarkers and potential hypothermic agents for chicks under thermal challenges (Han et al., 2018). In addition, Han et al. (2017, 2020) observed that amino acids ameliorated the effect of heat stress on the embryonic liver and brain by altering the metabolic amino acids of birds.

As a sulfur-containing amino acid, cysteine is synthesized from methionine by transsulfuration, where it condenses with serine to form cystathionine, which is then converted to cysteine by cystathionine Çystath (Stipanuk and Ueki, 2011; Vilar da Silva et al., 2020). The findings of Vilar da Silva et al. (2020) revealed that if cysteine metabolism is compromised, it could affect thermoregulation and thus feed efficiency and bird welfare (Vilar da Silva, et al., 2020). Cysteine is essential for protein structure and function and it is also a constituent of GSH, an antioxidant molecule (Mari et al., 2009). It is a precursor of several important enzymes for antioxidant protection (Jong et al., 2012). On the other hand, lysine ranks next to methionine as a limiting amino acid in poultry production (Kidd et al., 2013). Lysine is essentially involved in protein synthesis (Kheiri and Alibeyghi, 2017) and some active substances, including L-carnitine, are formed from lysine (Hull’ar et al., 2008). The observation of Huang et al. (2021) and Irani et al. (2015) indicated that dietary lysine improved animal antioxidant capacity. Moreover, it has been shown that the requirement for lysine increases during thermal stress (Han and Baker, 1993).

There is growing attention on the in ovo delivery of various substances during incubation and injection of antioxidants may be beneficial for the biochemical and physiological balances as well as enhancing the antioxidant status of the embryo and post-hatch growth (Salary et al., 2014; Elnesr et al., 2019; Elwan et al., 2019; Oke et al., 2021b). The perinatal phase is essential in chick’s development as they shift from using endogenous nutrients to using exogenous feed at this time (Ferket, 2012). Dysfunction of the antioxidant system inside the egg or in the body of the chicken results in lower hatchability and subsequent performance (Niu et al., 2009). As a result of their higher growth rate, the fast-growing present-day strains of broilers have higher metabolic demands and supplementation of nutrients before hatching might contribute to better growth and overcome the nutrient limitations of eggs (Foye et al., 2006). As avian embryos grow faster at the late stages of incubation, their nutritional needs increase. At this critical phase, in ovo administration of amino acids can be beneficial (Ebrahimi et al., 2017) as they are involved in thermoregulation and growth (Chowdhury et al., 2015). Additionally, Han et al. (2020) demonstrated that in ovo feeding of amino acids has been reported to improve broiler chickens subjected to acute stress thermotolerance. Since the level of lysine in the hatching eggs reduced as incubation advanced in the late phase (Zhu et al., 2019), administration of in ovo lysine may be beneficial to developing embryos at this stage. Ebrahimi et al. (2017) found that in ovo lysine enhanced the intestinal morphology, muscle weight and health status of broiler chickens. The metabolism of Sulphur-containing amino acids like cysteine can also be affected by heat stress in chicks (Ito et al., 2015).

Published data are scarce on the influence of in ovo feeding of cysteine plus lysine in ameliorating oxidative stress or heat stress of hatched chicks and their post-hatch performance. It was hypothesized that in ovo injection of cysteine and lysine may enhance the antioxidant constituents of embryos and hence alleviates the effect of stress on the chicks. Therefore, the aim of this study was to evaluate the influence of cysteine plus lysine on the physiological responses and post-hatch growth of thermally challenged broiler chickens in a tropical environment.

## 2 Materials and methods

### 2.1 Experimental procedure

A total of 2000 fertile eggs from a broiler breeder (Ross 308) flock (age 38 weeks) was obtained from a commercial farm. Between days 1 and 10, the fertile eggs were incubated using the optimal temperature and relative humidity conditions of 37.8°C and 55%, respectively. However, the temperature was increased to 39.6°C, 6 h per day, from embryonic day 10–17.5. Thereafter, the temperature of 37.5°C was used until hatching. On day 17.5, a total of 1,500 eggs with the evidence of living embryos were assigned to 6 treatments having five replicates of 50 eggs viz.: 0 (un-injected eggs) (UI), eggs injected with only 0.5 ml distilled water (DW), 3.5mg/egg cysteine (CY), 2mg/egg lysine (LY), 3.4 mg cysteine + 2 mg lysine (CLH) and 1.7 mg cysteine + 1 mg lysine (CLL) dissolved in 0.5 ml distilled water. Eggs with living embryos were injected with 0.5 ml of solution using a 24G hypodermic needle (25-mm long) through the holes in the shell above the air chamber, and the pinpoint hole was sealed with wax. As in the setter unit, eggs were transferred to hatching baskets arranged in the hatcher unit to match the arrangement of trays for each replicate of each treatment. Relative humidity and temperature of 65% and 36.7°C, respectively were used for hatching eggs in the hatcher.

### 2.2 Hatchability determination

The hatchability of fertile egg in each treatment was determined using the following formula:

$$\text{Hatchability}\left(\text{\%}\right)\text{:}=\frac{\text{Number of eggs hatched }}{\text{Number of eggs with living embroys}}\text{×}100$$ (1)

### 2.3 Evaluation of hatchling parameters

Immediately after hatching, the chick quality assessment was done as described by Tona et al. (2003), assessing the physical parameter including reflex, feathering condition down and appearance, eyes, the conformation of legs, navel area, yolk sac, and remaining membranes and yolk. At hatch, two chicks in each replicate were euthanized, and the relative weights of the liver, heart, yolk sac, gizzard, hatching muscles, intestinal weight, and yolk-free body weight will be recorded.

### 2.4 Post–hatch management

After hatch, the chicks were reared for 56 days of age according to the in ovo treatment to examine the effects of substrate injections on performance and heat tolerance. The chicks in each treatment were weighed, tagged, and replicated with similar weights and distribution. The birds were fed *ad libitum* with feed formulated to meet the nutrient requirement of broiler chicken recommended by National Research Council (1994) guidelines.

### 2.5 Data collection

#### 2.5.1 Growth performance

On a replicate basis, the feed intake and live body weights were evaluated throughout the experiment using a sensitive scale and mortality was monitored daily. The feed conversion ratio and weight gain were determined and adjusted for mortality. Feed conversion was calculated as the feed consumed and weight gain ratio.

#### 2.5.2 Blood analysis

Blood samples were collected through the jugular veins of 2 randomly selected birds per replicate into tubes containing EDTA and were analyzed for red blood cells, lymphocytes, white blood cells, packed cell volume, haemoglobin, monocytes, heterophil, eosinophils and basophil with the use of an automated analyzer (Hitachi 760; Hitachi Co., Tokyo, Japan).

Blood samples (about 2 ml) were also collected from birds per replicate and into serum tubes for serum biochemical indices. The samples were centrifuged for 15 min for the separation of serum and analyzed for total protein, glucose albumin, globulin, urea, creatine kinase, alanine aminotransferase, and aspartate aminotransferase using a biochemical analyzer (Hitachi, Japan). The globulin was calculated by subtracting the albumin from the total protein.

The concentrations of serum catalase, superoxide dismutase activity and malondialdehyde were analyzed using the commercial kits according to the instructions of the manufacturer (Jiangsu BaoLai Biotechnology Co., Yancheng, China).

#### 2.5.3 Intestinal morphology

At the end of the experimental period (56 days), ten birds per treatment, with body weight close to the treatment average body live weight, were selected for evaluation of carcass traits. Selected birds were subjected to 12 h of fasting prior to slaughtering. From the descending duodenum, middle jejunum, and ileum segment, approximately 4–5 cm of fragments were excised and fixed for 72 h in 10% neutral-buffered formalin after being flushed with distilled water. An examination with a light microscope was conducted by placing 5-μm sections of each paraffin-embedded sample on a glass slide, stained by hematoxylin-eosin. The morphological traits, including crypt depth, villus width, villus height and the ratio of villus height to crypt depth, were evaluated following the description of Allameh and Toghyani (2019). The villus surface area (VSA) was calculated as:

VSA = ½ x VW x VH x 2π.

Where: VH = Villus height, VW = Villus width, *π* = 3.14.

### 2.6 Statistical analysis

Data collected were subjected to analysis of variance using SAS (2008) in a Completely Randomized Design. Tukey’s honestly significant difference test was used to compare differences among treatments at *p* < 0.05.

## 3 Results

The influence of *in ovo* cysteine and lysine on the chick quality characteristics and the hatchability of broiler chickens is shown in Table 1. The hatchability of CY was higher than the other treatments. The lowest values were recorded in CLH. There was a similarity in the scores of the eyes, appearance, activity, navel area, remaining membrane, leg, and the remaining yolk. However, the chick weight of the CY was similar to those of CLL, LY, DW and UI but higher than that of CLH. The chick-egg ratio of the LY was not different from those of the other treatments but lower than CY. The yolk absorption of CY treatment group recorded a lower yolk absorption than those of the other treatments whose values were similar. The overall score of LY and CLL was similar and ranked higher than the rest of the treatments.

**TABLE 1:** Effect of *in ovo* cysteine and lysine on hatchability and chick quality at hatch.

Parameters	UI	DW	L Y	CY	CLH	CLL	SEM	*p* Value
Egg weight (g)	71.06	71.60	70.44	70.30	71.34	71.15	5.43	0.988
Hatchability (%)	79.25^b^	77.80^b^	80.15^b^	87.73^a^	58.59^c^	78.63^b^	1.911	0.0001
Chick weight (g)	42.26^ab^	42.62^ab^	42.12^ab^	43.50^a^	42.02^b^	42.55^ab^	1.78	0.00 1
Chick/egg Ratio	0.59^ab^	0.59^ab^	0.59^b^	0.62^a^	0.59^ab^	0.59^ab^	0.04	<0.001
Eye	10.00	12.00	14.00	12.00	12.00	12.00	5.92	0.657
Appearance	9.00	9.50	10.00	10.00	10.00	9.00	0.87	0.264
Activity	6.00	6.00	5.75	6.00	5.50	5.50	0.90	0.678
Navel area	10.50	9.00	12.00	9.00	9.00	9.00	3.00	0.456
Remaining membrane	10.00	8.00	11.00	11.00	10.00	10.00	2.95	0.643
Leg	12.00	16.00	12.00	12.00	12.00	16.00	2.90	0.544
Remaining yolk	15.00	11.00	15.00	15.00	11.00	16.00	2.67	0.608
Yolk absorption	12.00^a^	12.00^a^	12.00^a^	9.00^b^	12.00^a^	12.00^a^	1.26	0.004
Total scores	84.50^b^	83.50^b^	91.75^a^	84.00^b^	81.50^b^	92.00^a^	5.82	0.045

^abc:^ Means value within the row bearing different superscript differ significantly (p< 0.05). UI, Un-injected eggs; DW, eggs injected with Distilled water; LY, Eggs injected 2 mg lysine; CY, Eggs injected 3.4 mg cysteine; CLH, Eggs injected mixture of 3.4 mg cysteine plus 2 mg lysine; CLL, Eggs injected mixture of 1.7 mg cysteine plus 1 mg lysine.

The effect of in ovo cysteine and lysine on anatomical characteristics of day-old chicks is shown in Table 2. There was no difference in the weights of the liver, gizzard, heart and intestine. However, the weight of the hatching muscle in CLL chicks was similar to CY chicks but higher (*p* < 0.05) than those in the other treatments. The residual yolk of CLH, CLL and UI chicks was similar and higher than DW, LY and CY chicks.

**TABLE 2:** Effect of *in ovo* cysteine and lysine on anatomical characteristics of day-old chicks.

Parameters	UI	DW	L Y	CY	CLH	CLL	SEM	*p* Value
Liver (%)	1.36	1.29	0.92	0.96	1.32	1.44	0.177	0.2552
HM (%)	0.20^b^	0.17^bc^	0.17^bc^	0.34^ab^	0.11^c^	0.38^a^	0.057	0.0011
Gizzard (%)	3.26	3.46	2.41	1.80	2.71	3.26	0.415	0.2431
IW (%)	2.68	2.43	2.01	1.62	1.95	2.43	0.274	0.4334
Heart (%)	0.32	0.30	0.28	0.23	0.31	0.30	0.047	0.2630
Residual Yolk (%)	2.37^a^	1.01^b^	1.24^bc^	0.98^c^	2.75^a^	2.56^a^	1.853	0.0165

^abc:^ Means value within the row bearing different superscript differ significantly (p< 0.05). UI, Un-injected eggs; DW, eggs injected with Distilled water; LY, Eggs injected 2 mg lysine; CY, Eggs injected 3.4 mg cysteine; CLH, Eggs injected mixture of 3.4 mg cysteine plus 2 mg lysine; CLL, Eggs injected mixture of 1.7 mg cysteine plus 1 mg lysine; IW, intestinal weight.

Table 3 shows the effect of *in ovo* cysteine and lysine on plasma catalase, MDA, SOD of day-old chicks. The MDA of DW and UI chickens was similar and higher than birds in the other treatment groups. The levels in LY and CY birds were intermediate and higher than those of CLH and CLL. There was no difference in the catalase of the birds across the treatment groups. The serum SOD of CLL birds was comparable with that of CY but higher than those of the other treatments, whereas the SOD of UI was not different from that of DW but was lower than the others.

**TABLE 3:** Effect of *in ovo* cysteine and lysine on plasma catalase, MDA, SOD of day-old chicks.

Parameters	UI	DW	L Y	CY	CLH	CLL	SEM	*p* Value
MDA (nmol/ml)	3.304^ab^	4.451^a^	1.857^c^	2.021^c^	0.813^d^	0.757^d^	0.284	0.001
SOD (ng/ml)	2.490^d^	3.189cd	5.431^bc^	7.581^ab^	4.186^cd^	9.564^a^	0.553	0.0001
CAT(U/mml)	2.342	1.920	2.557	3.385	2.618	3.573	0.214	0.197

^abc:^ Means value within the row bearing different superscript differ significantly (p< 0.05). UI, Un-injected eggs; DW, eggs injected with distilled water; LY, Eggs injected 2 mg lysine; CY, Eggs injected 3.4 mg cysteine; CLH, Eggs injected mixture of 3.4 mg cysteine plus 2 mg lysine; CLL, Eggs injected mixture of 1.7 mg cysteine plus 1 mg lysine.

The total protein of the birds of LY, CY and CLH was not significantly different but higher than those of the UI and DW chicks (Table 4). However, the total protein of CLL birds was higher than those of the other treatments. There was no difference in the serum creatinine, AST, ALT, cholesterol, glucose and triglycerides of the chicks across the treatments. The serum albumin of CLH chicks was similar to those of CLL, CY, DW and UI chicks but higher than those of LY, which was higher than those of UI and DW. The serum globulin of CLL chicks was higher than those of CLH, CY and LY, which were also higher than those of UI and DW chicks. The haematological parameters of the chickens were comparable across different treatment groups (Table 5).

**TABLE 4:** Effect of *in ovo* cysteine and lysine on serum biochemical indices of day-old chicks.

Parameters	UI	DW	L Y	CY	CLH	CLL	SEM	*p* Value
Total protein (mg/dl)	8.07^c^	8.20^c^	8.83^b^	8.76^b^	9.03^b^	9.76^a^	0.137	0.0001
Creatinine (mg/dl)	0.70	0.73	0.76	0.76	0.73	0.73	0.50	0.647
ALT (U/L)	37.67	38.67	40.67	41.00	40.00	40.00	2.05	0.366
AST (U/L)	219.67	246.00	241.00	249.33	251.00	258.67	4.399	0.1509
Albumin (mg/dl)	3.26^ab^	3.37^ab^	3.20^b^	3.23^ab^	3.50^a^	3.27^ab^	0.031	0.0362
Globulin (mg/dl)	4.80^c^	4.83^c^	5.63^b^	5.53^b^	5.53^b^	6.50^a^	0.142	0.0001
ALB: GLB	0.68^a^	0.70^a^	0.57^bc^	0.58^bc^	0.63^ab^	0.50^c^	0.018	0.0002
Glucose (mg/dl)	324.33	363.33	359.67	366.33	361.67	350.00	20.33	0.081
Cholesterol (mg/dl)	208.00	234.67	237.33	241.33	240.00	243.67	16.84	0.065
Triglyceride (mg/dl)	91.00	95.33	94.67	96.00	93.33	86.33	8.03	0.754

^abc:^ Means value within the row bearing different superscript differ significantly (p< 0.05). UI, Un-injected eggs; DW, eggs injected with distilled water; LY, Eggs injected 2 mg lysine; CY, Eggs injected 3.4 mg cysteine; CLH, Eggs injected mixture of 3.4 mg cysteine plus 2 mg lysine; CLL, Eggs injected mixture of 1.7 mg cysteine plus 1 mg lysine.

**TABLE 5:** Effect of *in ovo* cysteine and lysine on haematology at hatch.

Parameter	UI	DW	L Y	CY	CLH	CLL	SEM	*p* Value
PCV (%)	28.00	27.76	26.67	23.50	23.00	29.67	1.17	0.600
HB (g/dl)	7.66	6.25	8.66	6.25	6.90	7.00	0.39	0.639
RBC (×10^6 ^/L)	2.10	1.75	1.49	1.81	1.48	2.65	0.56	0.062
WBC (×10^6 ^/L)	14.45	14.15	12.12	12.90	13.03	13.78	16.71	00.61
Platelet (10^3^/µL)	13.30	11.83	11.67	11.55	11.90	12.73	10.21	0.257
Lymphocyte (%)	57.00	58.33	51.67	55.00	57.67	62.00	5.30	0.299
Heterophil (%)	33.00	38.00	37.67	35.00	38.33	31.67	4.49	0.335
Eosinophil (%)	5.00	4.50	2.67	4.50	2.67	4.33	1.74	0.439
Basophil	0.67	0.33	1.00	0.50	0.33	0.33	0.50	0.661

UI, Un-injected eggs; DW, eggs injected with Distilled water; LY, Eggs injected 2 mg lysine; CY, Eggs injected 3.4 mg cysteine; CLH, Eggs injected mixture of 3.4 mg cysteine plus 2 mg lysine; CLL, Eggs injected mixture of 1.7 mg cysteine plus 1 mg lysine.

Table 6 shows the influence of *in ovo* cysteine and lysine on performance broiler chickens. The weights of the day-old chicks of UI and DW were not significantly different but lower than those of CY (Table 6). However, the weights of chicks were comparable with those of LY, CLH and CLL. The final weights of CLL chickens were similar to those of LY but higher (*p* < 0.05) compared to the other treatments. The feed intake of the CY and CLL and LY chickens was similar and higher (*p* < 0.05) than birds of the other treatment groups. The feed conversion ratios of the chickens were not different from one another.

**TABLE 6:** Influence of *in ovo* cysteine and lysine on performance broiler chickens.

Parameters	UI	DW	L Y	CY	CLH	CLL	SEM	*p* Value
Initial weight (g)	42.45^b^	42.30^b^	42.57^ab^	43.40^a^	42.65^ab^	42.91^ab^	0.094	0.0082
Final weight (g)	2760.0^c^	2720.0^c^	3180.0^ab^	3137.0^b^	2825.0^c^	3285.0^a^	31.137	0.0001
weight gain (g)	2717.55^c^	2677.70^c^	3137.42^ab^	3093.59^b^	2782.36^c^	3242.09^a^	31.109	0.0001
Feed intake (g)	5922.05^b^	6086.78^b^	6837.53^a^	6725.45^a^	5840.51^b^	6746.69^a^	68.385	0.0001
FCR	2.18	2.27	2.18	2.17	2.10	2.12	0.019	0.0890

^abc:^ Means value within the row bearing different superscript differ significantly (p< 0.05). UI, Un-injected eggs; DW, eggs injected with Distilled water; LY, Eggs injected 2 mg lysine; CY, Eggs injected 3.4 mg cysteine; CLH, Eggs injected mixture of 3.4 mg cysteine plus 2 mg lysine; CLL, Eggs injected mixture of 1.7 mg cysteine plus 1 mg lysine.

Table 7 shows the effect of in ovo cysteine and lysine on the intestinal morphology of broiler chickens. The duodenal villus heights of the birds of CLL were not significantly different from those in CLH but higher than those of the other treatment groups, whereas the villus height of the birds of CLH was similar to those of LY but higher than those of UI, DW and CY. The duodenal villus width of the CY birds was not different from those of LY and CLL but higher than those of DW and UI. The villus surface area and villus height/crypt depth ratio of CLL chickens were significantly higher than the birds in the other treatment groups. The jejunal villus width of the birds was not different. However, the jejunal villus heights of the birds of CLL were higher than those of the birds of the other groups and the values recorded in CLH birds were higher than those of UI and DW groups. The jejunal crypt depth of CLL, UI and DW was not different from those of CLH and CLL birds and was significantly higher than that of LY. The values in LY were similar to those recorded in CLH and CLL. The ratio of villus height to crypt depth of the birds in DW, UI, and CLH was comparable to those of CY but significantly shallower than the others. The surface area of CY birds was not different from the CLL chickens but higher than the other treatment groups. The ileal villus height in the birds of CLL was not different from that of CLH and LY but higher than those of the other treatments. The ileal villus width of the birds of UI, LY, CY was similar to that of DW but narrower than that of CLL. The crypt depth of the UI chickens was similar to those of DW, and CLH but deeper than LY, CY and CLL chickens. The villus height/crypt depth ratio in the ileum of the CLL chickens was similar to those of CLH and CY but higher than the other groups. The ratios in UI and DW birds were the least. The villus surface area of the birds with CLL was higher than the rest of the treatment groups, which were not significantly different.

**TABLE 7:** Effect of *in ovo* cysteine and lysine on intestinal morphology of broiler chickens.

Parameters	UI	DW	L Y	CY	CLH	CLL	SEM	*p* Value
**Duodenum**								
VH (µm)	364.23^d^	342.12^d^	471.58^bc^	399.75^cd^	544.23^ab^	583.65^a^	20.24	0.0001
VW (µm)	74.64^b^	82.78^b^	103.35^ab^	139.13^a^	87.80^b^	124.07^ab^	6.29	0.0046
CD (µm)	75.95^c^	93.85^bc^	98.18^abc^	114.20^ab^	120.45^a^	81.75^c^	3.862	0.0001
VH:CD	4.93^b^	3.64^b^	4.82^b^	3.54^b^	4.59b	7.15^a^	0.278	0.0001
VSA (mm^2^)	0.85^c^	0.88^c^	0.15^b^	0.17^b^	0.15^b^	0.23^a^	0.018	0.0001
**Jejunum**								
VH (µm)	338.37^c^	350.10^c^	447.48^bc^	535.47^ab^	377.80^c^	644.45^a^	24.73	0.0029
VW (µm)	106.42	102.40	99.28	131.67	109.68	87.80	5.729	0.3932
CD (µm)	107.65^a^	99.27^a^	73.02^b^	112.55^a^	93.18^ab^	92.50^ab^	3.341	0.0018
VH:CD	3.16^c^	3.54^c^	6.13^ab^	4.81^bc^	4.20^c^	6.99^a^	0.319	0.0001
VSA (mm^2^)	0.11^b^	0.11^b^	0.14^b^	0.22^a^	0.13^b^	0.18^ab^	0.097	0.0007
**Ileum**								
VH (µm)	338.45^cd^	276.55^d^	500.25^abc^	429.45^bcd^	555.05^ab^	651.00^a^	29.592	0.0001
VW (µm)	89.07^b^	98.28^ab^	93.00^b^	82.33^b^	76.63^b^	138.77^a^	5.417	0.0029
CD (µm)	127.75^a^	96.25^ab^	86.70^b^	64.27^b^	93.43^ab^	83.92^b^	5.026	0.0022
VH:CD	4.07^c^	2.87^c^	5.76^b^	6.61^ab^	6.18^ab^	7.77^a^	0.362	0.0001
VSA (mm^2^)	0.95^b^	0.85^b^	0.155^b^	0.12^b^	0.13^b^	0.28^a^	0.016	0.0001

^abc:^ Means value within the row bearing different superscript differ significantly (p < 0.05). UI, Un-injected eggs; DW, eggs injected with distilled water; LY, Eggs injected 2 mg lysine; CY, Eggs injected 3.4 mg cysteine; CLH, Eggs injected mixture of 3.4 mg cysteine plus 2 mg lysine; CLL, Eggs injected mixture of 1.7 mg cysteine plus 1 mg lysine; VW, villus width; VH, villus height; CD, crypt depth; VSA, villus surface area.

## 4 Discussion

This study intended to evaluate the effects of cysteine plus lysine on the hatching, physiological responses and post-hatch growth of broiler embryos subjected to heat stress during incubation in a tropical environment. The improved hatchability obtained in the CY group in this study suggests that the in ovo feeding of cysteine enhanced the embryos’ antioxidant status. The administration of exogenous substances during incubation has been reported to reduce free radical production, which hampers the cellular membranes of the developing embryos, thereby leading to better utilization of lipid for the production of energy needed for hatching (Noh et al., 2013). Similar to this observation, Kalantar et al. (2019), demonstrated that in ovo injection of Coenzyme Q10 enhanced the hatchability of broilers. The in ovo feeding of lysine alone did not positively affect the hatchability. This result corroborates the recent findings of Zhu et al. (2019), who did not record any improvement in hatchability of pigeons treated with in ovo feeding of lysine. The lower hatchability recorded in the CLH group in this study suggests that the mixtures of these amino acids at this level were not beneficial to the embryos. The similarity in the hatchability of the chicks from in ovo lysine and the lower level of combination of cysteine and lysine with the control groups indicates that these inclusion levels were not detrimental to the embryos. In contrast to our findings, Coskun et al. (2018) indicated that in ovo injection of lysine had a higher hatchability compared to the control group. The discrepancy may be attributed to amino acid imbalance. Several factors such as insufficient nutrients in the egg, lethal genes, shell thickness, egg weight, age of the breeder flock, nutrition, health, strain, and incubation conditions can influence hatchability (King'Ori, 2011). The higher hatchability recorded in the in ovo cysteine suggests that this in ovo feeding remediated these variables. The lower hatchability recorded in the CLH group in this study indicates that the ratio of the amino acid combination was not ideal for the embryos. The metabolism of embryos can be significantly influenced by the amino acid ratio (Kheiri and Alibeyghi, 2017). Moreover, the slight higher day-old chick weights observed in the in ovo feeding of cysteine in the present study suggests that the antioxidant status of the embryos was enhanced. This observation corroborates the findings of Elwan et al. (2019), who indicated that the in ovo feeding of the mixture of methionine and cysteine improved the day-old chick weights of broilers.

The in ovo feeding of lysine showed a higher total score for the quality characteristics of the chicks in the present study. This may suggest that the nutrient metabolism of chicks in this group was more enhanced by the administration of the amino acid (Tona et al., 2004). Similarly, N’nanle et al. (2017) reported that in ovo feeding of Moringa oleifera improved the total score for chick quality in broilers. Moreover, the better yolk absorption recorded in the in ovo feeding of cysteine treatment in the present study aligns with the higher hatchability of the chicks. This observation indicates that an increased lipid metabolism led to a higher production of energy needed for hatching events. There was no pronounced difference in the chick weights at hatch in the present study. However, Bhanja and Mandal (2005) demonstrated that a combination of amino acids in ovo feeding improved the chicks’ weight. The discrepancy may be due to the number and the types of amino acids combined.

The lower residual yolk of the CY chicks than the control group in the present study indicates that the amino acids favoured energy utilisation to support hatching. Using glycogen is essential during the hatching process (Moran, 2007). During pipping, the chorioallantoic membrane is separated, limiting oxygen delivery and lipid breakdown. From this point on, the hatching muscle relies only on glucose from glycogen stores (Freeman, 1969). This observation can explain the higher hatchability recorded in the CY treatment group. A higher level of nutrients may cause hyperplasia in myofibers, which leads to increased muscle production because chicks’ myofibers are already formed at hatching (Smith, 1963). The relative higher hatching muscle of CLL chicks, similar to the CY group in the present study suggests that the in ovo feeding of these amino acids had a positive effect on the chicks.

Various antioxidant factors in the body are responsible for eliminating free radicals in the body (Zhang et al., 2017; Bai et al., 2019; Zhang et al., 2020). The plasma MDA and SOD of the birds treated with in ovo feeding of cysteine was better than the chicks of the control groups in the present study. When birds are under stress, the demand for Sulphur-containing amino acid-like cysteine increases since it is required to synthesise GSH in order to mitigate the damage caused by OS induced by HS (Brosnan, 2006; Bunchasak, 2009; Wu, 2009). The amino acids have a high antioxidant capability, keep free radicals in check and maintain a healthy equilibrium in the body (Bin et al., 2017). The results in this study demonstrated that the in ovo feeding of the amino had positive effects on the birds. Further, the lower combination of in ovo feeding of lysine and cysteine was better than their separate injections, suggesting a beneficial synergistic effect of the amino acids. Similar to our findings, Elnesr et al. (2019) demonstrated that in ovo feeding of Sulphur-containing amino improved plasma MDA and superoxide dismutase of broiler chicks.

The higher feed intake observed in the chickens from in ovo feeding of lysine, cysteine and a mixture of lysine and cysteine in this study affirms the observation of Uni and Ferket (2004), who demonstrated that in ovo feeding may stimulate appetite in chickens. The higher feed intake of the birds suggests that the amino acids enhanced the gut health and the production of its hormones, thereby leading to increased feed utilization in the birds (Gao et al., 2017). In ovo feeding of other amino acids such as arginine and threonine have been documented to improve feed intake (Tahmasebi and Toghyani, 2016; Gao et al., 2017). We recommend further findings on the effects of in ovo feeding on the gut health and ghrelin and leptin production in birds. The higher weights of the birds from in ovo lysine in the present study are in tandem with the observation of Ebrahimi et al. (2017), who indicated that in ovo feeding of lysine increased the final body weights of broiler chickens. The higher final weights of the birds from the lower level of the cysteine and lysine (CLL) mixture in this study suggest that this was more beneficial to the birds than the higher combinations (CLH). Similar to our observation, Bhanja et al. (2012) demonstrated that the final weights of broiler chickens subjected to in ovo feeding of methionine and lysine were improved. Interestingly, although the initial chick weight of CY birds was higher at hatch; however, this superiority faded off as the birds grew older and the CLL birds had a higher catch-up growth with resultant higher final weights.

The pathological and physiological conditions of animals can be assessed through their haematological parameters (Esonu et al., 2001; Scheele et al., 2003). The values obtained were within the normal range (Campbell, 2012). The similarity in the haematological parameters of the birds in this study suggests that the in ovo feeding did not have a deleterious effect on the chicks. This observation conforms with the findings of Sogunle et al. (2019), who showed that in ovo feeding of some amino acids did not affect the haematology of broilers.

Biochemical indices may reflect the health condition of an animal (Toghyani et al., 2010; Zhang et al., 2018). The total protein can indicate protein utilization and absorption, while albumin in plasma is a good indicator of the body’s nutritional condition (Schmilovitz-Weiss et al., 2006). It is noted that the total protein of the birds was higher than those in the control groups. This indicates that the birds benefitted from the in ovo feeding of the amino acids. As an essential amino acid, lysine plays a vital role in the body’s physiological metabolism, mostly in protein synthesis rather than metabolic processes (Kheiri and Alibeyghi, 2017). The in ovo feeding of lysine has been shown to enhance blood metabolites (Ebrahimi et al., 2017). Interestingly, the low-dose of the mixture of lysine and cysteine in the present study elicited higher total protein than their separate doses. This suggests that there was a favourable synergy between the amino acids. A similar trend was also observed in the globulin of the birds. Globulin essentially comprises several constituents like immunoglobulin and complement, which can be a biomarker of the body’s immunological activity.

The development of the small intestine is very dependent on late-term incubation (Ohta and Kidd, 2001; Uni et al., 2003). The use of in ovo feeding can remedy the issue of delayed feeding, which can occur due to the delivery process from hatcheries to commercial farms after hatching (Ferket, 2012) and this could impede the early development of the gastrointestinal tract and post-hatch growth of chicks (Geyra et al., 2002). As a result, IOF of exogenous nutrients may help chickens’ gut health and enhance their feed intake, resulting in improved growth performance (Zhao et al., 2017). The birds’ higher duodenal, jejunal and ileal villi heights and surface area from the in ovo feeding of the low-dose of cysteine and lysine (CLL) in the present study demonstrate the beneficial effect of these amino acids on the developing embryos. Villi size and height are critical for intestinal function because higher villi height implies increased intestinal surface area and enhanced nutritional absorption (Soltan, 2009; Izadi et al., 2013). This improved morphology could explain the improved growth performance of the CLL chickens, as a higher surface area would aid nutritional digestion and assimilation, increasing body weight (Tako et al., 2004). The shape of intestinal villi has been connected to growth, with longer villi being linked to enhanced body weight gain (Maneewan Yamauchi, 2004). Similar to our findings, Zhu et al. (2019) reported that in ovo feeding of lysine improved the intestinal morphology of pigeons, while the author also reported that the mixture of methionine and lysine improved the digestibility of nutrients in chickens (Tako et al., 2005).

The results of the present result are in concomitance with the earlier findings of Ebrahimi et al. (2017), who indicated that in the ileum, jejunum and duodenum, in ovo lysine injection enhanced intestinal morphology, including villus height and villus height/crypt depth ratio while decreasing crypt depth. There is, however, a scarcity of published data on the in ovo feeding of cysteine. The lack of pronounced improvement in the intestinal traits of the chickens from in ovo cysteine in this study suggests that its effects faded with the age of the birds because the amino acid enhanced the perinatal traits. In contrast to the findings in the present study, Bhanja and Mandal (2005) indicated the in ovo feeding of the mixture of amino acids, including lysine, methionine and cysteine or lysine and arginine did not influence the intestinal morphology of broilers.

To summarize, in ovo feeding of cysteine or lysine improved of the antioxidant status of broiler chicks. This study revealed that in ovo feeding of a low-dose mixture of cysteine and lysine on thermally-challenged chicken embryos could be considered biphasic as the effect was less pronounced at the perinatal phase of growth but improved the intestinal morphology of broiler chickens at a later stage. The improvement in the birds’ gut health translated to better growth performance. In ovo feeding of cysteine alone was more effective on the hatchlings and the effects faded at the market age.

## Data Availability

The raw data supporting the conclusions of this article will be made available by the authors, without undue reservation.
